# Silicon nanoparticles and indole butyric acid positively regulate the growth performance of *Freesia refracta* by ameliorating oxidative stress under chromium toxicity

**DOI:** 10.3389/fpls.2024.1437276

**Published:** 2024-08-02

**Authors:** Muhammad Ahsan, Emanuele Radicetti, Aftab Jamal, Hayssam M. Ali, Mateen Sajid, Abdul Manan, Ali Bakhsh, Muhammad Naeem, Jawad Ahmad Khan, Mohammad Valipour

**Affiliations:** ^1^ Department of Horticultural Sciences, The Islamia University of Bahawalpur, Bahawalpur, Pakistan; ^2^ Department of Chemical, Pharmaceutical and Agricultural Sciences (DOCPAS), University of Ferara, Ferrara, Italy; ^3^ Department of Soil and Environmental Sciences, Faculty of Crop Production Sciences, The University of Agriculture, Peshawar, Pakistan; ^4^ Department of Botany and Microbiology, College of Science, King Saud University, Riyadh, Saudi Arabia; ^5^ Department of Horticulture, Ghazi University, Dera Ghazi Khan, Pakistan; ^6^ Department of Plant Breeding and Genetics, Ghazi University, Dera Ghazi Khan, Pakistan; ^7^ Department of Pharmacy, Shah Abdul Latif University Khairpur, Khairpur, Pakistan; ^8^ Department of Engineering and Engineering Technology, Metropolitan State University of Denver, Denver, CO, United States

**Keywords:** antioxidants, cut flower, heavy metal, lipid peroxidation, nanotechnology, photosynthesis

## Abstract

Chromium (Cr) toxicity hampers ornamental crops’ growth and post-harvest quality, especially in cut flower plants. Nano-enabled approaches have been developing with phenomenal potential towards improving floricultural crop production under heavy metal-stressed conditions. The current pot experiment aims to explore the ameliorative impact of silicon nanoparticles (Si-NPs; 10 mM) and indole butyric acid (IBA; 20 mM) against Cr stress (0.8 mM) in *Freesia refracta*. The results showed that Cr stress significantly reduced morphological traits, decreased roots-stems biomass, abridged chlorophyll (14.7%) and carotenoid contents (27.2%), limited gas exchange attributes (intercellular CO_2_ concentration (*Ci*) 24.8%, stomatal conductance (*gs*) 19.3% and photosynthetic rate (*A*) 28.8%), condensed proline (39.2%) and total protein (40%) contents and reduced vase life (15.3%) of freesia plants by increasing oxidative stress. Contrarily, antioxidant enzyme activities, MDA and H_2_O_2_ levels, and Cr concentrations in plant parts were remarkably enhanced in Cr-stressed plants than in the control. However, foliar supplementation of Si-NPs + IBA (combined form) to Cr-stressed plants increased defense mechanism and tolerance as revealed by improved vegetative and reproductive traits, increased biomass, photosynthetic pigments (chlorophyll 30.3%, carotenoid 57.2%) and gaseous exchange attributes (*Ci* 33.3%, *gs* 25.6%, *A* 31.1%), proline (54.5%), total protein (55.1%), and vase life (34.9%) of metal contaminated plants. Similarly, the improvement in the activities of peroxidase, catalase, and superoxide dismutase was recorded by 30.8%, 52.4%, and 60.8%, respectively, compared with Cr-stressed plants. Meanwhile, MDA (54.3%), H_2_O_2_ (32.7%) contents, and Cr levels in roots (43.3), in stems (44%), in leaves (52.8%), and in flowers (78.5%), were remarkably reduced due to combine application of Si-NPs + IBA as compared with Cr-stressed nontreated freesia plants. Thus, the hypothesis that the synergistic application of Si-NPs + IBA will be an effective approach in ameliorating Cr stress is authenticated from the results of this experiment. Furthermore, the study will be significant since it will demonstrate how Si-NPs and IBA can work synergistically to combat Cr toxicity, and even when added separately, they can improve growth characteristics both under stressed and un-stressed conditions.

## 1 Introduction

Heavy metals (HMs) constitute significant abiotic stressors that exert harmful impacts on plant development, crop yield, and overall agricultural sustainability worldwide (Wei et al., 2023). Concentrations of HMs and their bioavailability in the environment can potentially increase due to both anthropogenic and geogenic sources, including rock weathering, forest fires, industrial wastes, pesticides, paints, and smelting processes (Chen et al., 2022; Sarim et al., 2022; Liu et al., 2023). Amongst these, chromium (Cr) is one of the most toxic HM due to its bio-accumulative nature and longer shelf life (Ahmad et al., 2022). In various studies, it has been observed that exposure of plants to Cr, reduces growth performance, decreases plant biomass, disrupts the photosynthetic apparatus, interferes with antioxidant enzyme activities, and hampers the uptake of minerals and water (Habiba et al., 2019; Lopez-Bucio et al., 2022; Ahammed et al., 2023). Furthermore, Cr toxicity activates extreme reactive oxygen species (ROS) production in plant tissues, thus infuriating oxidative stress, disturbing the plants’ redox balance (Ashraf et al., 2022). Stress induced by Cr toxicity is credited to a complex series of metal relations with the genetic mechanisms, signal transduction, and cellular macromolecules (Sharma et al., 2020). Anjum et al. (2017) documented that Cr stress also plant growth by encouraging ultrastructural changes of the chloroplast and cell membrane, stimulating leaf chlorosis, decreasing pigment color, disturbing cells of the roots, damaging mineral nutrition and water relations, influencing assimilation of nitrogen and transpiration, and by changing the activities of enzymes. Therefore, efficient novel approaches are essential for the remediation of Cr-polluted soils to ensure sustainable agricultural crop production.

Nanotechnology has emerged as a cost-effective and eco-friendly technology with great potential for reclaiming soil polluted with Cr toxicity. It offers numerous benefits, including improvements in plant growth, enhanced nutrient use efficiency, and alleviation of HMs stress (Prakash et al., 2022). Nanoparticles (NPs) have attracted a lot of attention due to their efficient ability to address phytotoxicity through their changeable and manageable surface properties (Feng et al., 2022; Liu et al., 2023). The NPs may aid in seed germination, reduce nutrient losses, mitigate diseases, and enhance plant productivity (Farooq et al., 2022). NPs reduce the accrual of hydrogen peroxide and malondialdehyde, stimulate the expression of stress-linked proteins, regulate water and nutrient absorption, and maintain the stability of the membrane (Rasheed et al., 2022). Hashem et al. (2021) reported that NPs have tendency to penetrate the chloroplast of the plants and reach the reaction center of photosystem-II and stimulate the electron transmission and light absorption in chloroplasts, thus enhancing efficacy of photosynthesis and plant growth. Silicon (Si) and Si-NPs have emerged as significant metalloids with various positive impacts on plants (Manivannan et al., 2023). More recently, Si-NPs have been shown to enhance the tolerance of various plants to HM stress by managing reactive oxygen species (ROS), photosynthetic processes, and antioxidant enzyme activities (Asgher et al., 2023; Huang et al., 2024). Furthermore, Meena et al. (2022) reported that Si-NPs can be effectively used to mitigate HM pollution in crops due to their extensive potential for HM alleviation. Previous studies have indicated that Si-NPs regulate antioxidant enzyme activities to trigger the defense system (Fatemi et al., 2021; Manzoor et al., 2023). Si-NPs show significant potential in reducing Cr toxicity by decreasing Cr uptake from the soil and enhancing enzyme activities (Sharma et al., 2022). Bhat et al. (2021) reported that Si-NPs may absorb Cr onto their surface or enter plant tissues, playing valuable roles. Additionally, Si-NPs assist in the strengthening the physical plant barriers, enhancing the anti-stress compounds’ productivity, and stimulating the expression of genes linked with defense (Wang et al., 2022). Khan et al. (2023) found that supplementation of Si-NPs under abiotic stresses raises the amino acids and soluble carbohydrates accrual in the fluid of xylem while maintaining osmosis.

Furthermore, auxins are a group of phytohormones that influence plants’ growth and responses to the environment (Tognetti et al., 2012). Besides, naturally occurring plant hormones, plants can also receive them exogenously (Sharma et al., 2022). These hormones are absorbed by various transporters and play a role in reducing stress caused by HMs. Various experiments have explored the impacts of indole acetic acid (IAA; natural auxin) on mineral nutrition and antioxidant defense mechanisms under heavy metal stress (Bashri and Prasad, 2016; Khan et al., 2019). However, IAA is not suitable as a fertilizer component due to its low stability and quick degradation (Cakmakçı et al., 2020). In contrast, indole butyric acid (IBA) is more stable than IAA. The stability of IBA, along with other fertilizers and bio-stimulants, depends on factors such as the level of oxygen, exposure to light, and heat (Kaczmarek et al., 2020).

The intrinsic contents of IBA are shown to be affected by heavy metal toxicity (Sharma et al., 2022). It was reported that exogenous supplementation of auxins and Si-NPs under abiotic stress on plants may significantly stimulate the defense response regulating growth (Kim et al., 2016). Furthermore, Si-NPs plays a role in controlling auxin concentrations within plants, while IBA alleviates heavy metal stress (Siposova et al., 2023). Sytar et al. (2019) stated that intrinsic auxin concentration may decrease under Cr stress, but Si-NPs not only help to regulate auxin levels but also improved the plant tolerance against heavy metal toxicity and therefore avert growth inhibition.

Freesia (*Freesia refracta*) is a specialty cut flower ornamental plant belonging to the family Iridaceae. This South African-origin cut flower has more than 300 cultivars worldwide, featuring flowers in shades of yellow, lavender, violet, pink, and golden hues. Extracts from freesia are commonly used in cosmetics, detergents, and candles due to their attractive fragrance (Ehrich et al., 2009). Economically, it ranks among the top ten cut flowers in global floricultural markets, and its demand is increasing (Ahmad et al., 2019). Freesia cut flowers are sensitive to ethylene which reduces vase life (Ahmad et al., 2019) while ethylene production increased due to reduced proline and protein contents under abiotic stresses including heavy metals. The supplementation of Si-NPs and auxin increased proline and total protein levels under heavy metal conditions (Sharma et al., 2022), which decreased ethylene levels and extended the post-harvest life of cut flower crops including freesia.

As nanomaterials can be prepared by eco-friendly and green methods, that can increase agriculture potential in the developing countries worldwide (Huang et al., 2015). Furthermore, nanoparticles minimize the amount of damaging chemicals (i.e., insecticides) that pollute the environment as well as reduce input costs. While the impacts of various NPs and IBA on plants treated with heavy metals have been studied independently, their synergistic effect on Cr-stressed plants still requires experimental attention. To investigate this aspect, we hypothesized that Si-NPs and IBA, and their combination is an effective approach for alleviating Cr stress in *Freesia refracta* plants in terms of positive impact on morphological attributes, biomass, photosynthesis, antioxidant enzymes, oxidative stress, accrual of the metal and the vase life of the elegant cut flowering plant. Nanoparticle use in metal-hoarded growing conditions will be better understood by evaluating the impact of NPs on elegant ornamental freesia under Cr toxicity.

## 2 Materials and methods

### 2.1 Plant growth conditions

A pot experiment was executed under lath house conditions at the research area of the Department of Botany, University of Education, Lahore, Pakistan, during 2021-2022. Uniform-sized corms of *Freesia refracta* were obtained from a local nursery and treated with Topsin-M fungicide at 2 g L^-1^ for eight minutes. After air drying under shade, the corms were planted in plastic pots of 18 inches filled with sandy clay loam soil (sand 42%, silt 30%, clay 28%). The electrical conductivity of the soil was 3.67 dSm^-1^ and pH was 7.7. The nutritional status of the soil was: nitrogen, 102 g kg^-1^ of soil, phosphorus, 12.37 g kg^-1^, potassium 154.6 g kg^-1^, and Cr contents were not detected. The corms were planted individually in each pot which was irrigated every two to three days. The mean light duration was about 10-11 h each day, with a mean temperature of 36 ± 3°C and mean relative humidity was about 64-70% throughout the experimental period.

### 2.2 Experimental treatments and layout

The current study comprised seven treatments. The treatment combinations were control (only tap water for irrigation), Cr stress (0.8 mM), Si-NPs (10 mM), IBA (20 mM), Si-NPs (10 mM) + Cr (0.8 mM), IBA (20 mM) + Cr (0.8 mM), Si-NPs (10 mM) + IBA (20 mM) + Cr (0.8 mM). These concentrations were prepared on a per-liter basis in the nutrient solution. There were eight plants in each treatment which were replicated five times (a total of 40 pots in each treatment), for a total of 280 freesia plants. These potted plants were arranged according to completely randomized design settings. Si-NPs (catalog # NC2820497) were purchased from an American-based company (Sigma-Aldrich, USA) with 99.8% purity with a particle size of <100 nm. The source of hexavalent Cr was potassium dichromate (Sigma-Aldrich, USA). After two weeks of corm sprouting, the hexavalent Cr treatment was supplemented to plants in Hoagland nutrient solution in the form of K_2_Cr_2_O_7_ salt solution. Cr-stressed plants after one week of vegetative growth were supplemented with Si-NPs, IBA, and Si-NPs + IBA foliar spray to respective freesia plants (five sprays on alternate days).

### 2.3 Characterization of Si-NPs

The solution of 10 mM Si-NPs was prepared by dissolving 600.8 mg of manufactured Si-NPs for 30 min in distilled water (1 L) by utilizing ultra-sonication by an ultrasonicator at 10 MHz for 40 min, resulting in a partially homogenous solution. Zeta potentials and hydrodynamic diameters were assessed by dynamic light scattering (DLS) with the help of Malvern Zetasizer Nano ZS (Worcestershire, UK). Measurements of pH and zeta potential were made in nutrient solutions at pH 5.8. The surface morphology, composition, and phase purity were evaluated using a scanning electron microscope (SEM) (TM1000, Hitachi, Japan) and by UV-Vis spectrophotometer (Specord 200 Plus).

### 2.4 Determination of vegetative and reproductive growth attributes

Days to flower emergence were counted from the corm sowing date while the number of flowers per stem was counted at the final stage of the experiment. The number of leaves per plant and leaf area was measured by using a leaf area meter (LICOR-3000C, Lincon, NE, USA). Plants were uprooted from the pots and washed under clean tap water. The roots and shoots were segregated with a sharp knife for root and shoot length measurements which were recorded by measuring tape. An electrical balance was used for root and shoot fresh weight, whereas, the dry weight of separated plant parts was recorded after 72 h by adjusting the electric oven temperature to 70°C (Ahsan et al., 2023).

### 2.5 Determination of SPAD chlorophyll and carotenoid contents

Leaf chlorophyll contents were estimated with the help of SPAD-502 (Konica, Minolta Sensing, Inc., Japan) by following the methodology described by Ling et al. (2011). Fully expanded topmost leaves were selected for measurement of SPAD values. Mean SPAD values measured from eight readings including the four readings each on both sides of the mid-rib were utilized as suggested by Leon et al. (2007). For determination of carotenoid (CAR) contents, a fresh freesia leaf sample (1.0 g) was collected and chopped into 5 mm pieces. In 10 mL of acetone (80%), the extraction was done overnight at 4°C for estimation of CAR by following the procedure of Davies (1976).

### 2.6 Gas exchange measurements

To estimate the gas exchange parameters i.e. intercellular CO_2_ concentrations (*Ci*), stomatal conductance (*gs*), and photosynthetic rate (*A*) were determined with the help of a portable open-flow gas exchange system viz. CIRAS-3 (PP system, Amesbury, USA). These measurements were recorded under clear sunlight (between 10:00 to 11:00 am) from fully opened upper leaves. The adjustment of the leaf chamber was done by following the documentation of Shehzad et al. (2020).

### 2.7 Measurements of proline and total protein contents

Proline contents were estimated by taking a sample (1.0 g) from the fresh leaf of freesia plants, homogenized in 5 mL aqueous sulfosalicylic acid (3%), as suggested by Ahmad et al. (2008) by using a spectrophotometer (Jenway, Staffordshire, UK). Total protein contents were determined from freshly harvested leaf samples with the help of a spectrophotometer at an absorbance of 595 nm according to a procedure suggested by Bradford (1976).

### 2.8 Assay of antioxidant enzyme activity

Fresh leaf sample (1.0 g) was normalized in dithiothreitol (DTT) and phosphate buffer (50 mM) and centrifuged for 20 min at 4°C and 12000 × *g* as proposed by Ngoc et al. (2022). The supernatant was detached, and the readings of the antioxidant enzyme activities were recorded with the help of a spectrophotometer (Jenway, Staffordshire, UK). At 470 nm absorbance, the peroxidase (POD) contents were recorded. At 240 nm absorbance, Tewari et al. (2013) methodology was adopted with minor changes for catalase (CAT) determination. The superoxide dismutase (SOD) activities were recorded at an absorbance of 560 nm as suggested by Njie et al. (2022) with minor modifications.

### 2.9 Measurements of hydrogen peroxide and malondialdehyde

The contents of H_2_O_2_ in freesia leaves were determined by taking a fresh leaf sample (1.0 g) and grinding with an ice-cooled mortar with 5 mL (*w/v*) of 0.1% trichloroacetic acid (TCA). The mixture of 10 mM phosphate buffer (pH 7.0) and 5 mM phosphate iodide was added to the supernatant and centrifuged at 12000 × *g* for 15 min. The blend optical density was noted at 390 nm after vortexing, the concentration was assessed against aqueous H_2_O_2_ (30%) standard as documented by Das et al. (2024). The MDA contents were recorded to assess the extent of lipid peroxidation by taking fresh leaf sample (1.0 g) and crushed with phosphate buffer solution (65 mM; pH 6.5) and with TCA 0.1% (*w/v*). The supernatant, after centrifugation for 10 min at 12000 × *g* at 4°C, was collected from reaction mixture and mixed with a mixture of thiobarbituric acid (0.5%) and TCA (20%). From After utilizing thiobarbituric acid (TBA) reaction. The mixture was incubated for 30 min at 95°C and cooled for 1h in ice bath. For 5 min, the centrifugation at 10,000× *g* and supernatant read at 532 nm and 600 nm, respectively, as suggested by Pal et al. (2024).

### 2.10 Determination of Cr concentrations

Concentrations of Cr in roots, stems, leaves, and flowers of freesia plants were determined by taking dry samples (0.5 g) and digested separately for 48 h at 75°C with an HNO_3_:HClO_4_ by following the methodology of Singh et al. (2023). Every sample was filtered and diluted to 60 mL with distilled water. The Cr levels in the mentioned parts of freesia were estimated by using an atomic absorption spectrophotometer (AAS-ZEEnit 700P, Analytik Jena, Germany).

### 2.11 Vase life estimation

In the early hours of sunrise (6:30-7:30 am), equally graded spikes of freesia were harvested manually by the sterilized blade. These spikes were vertically put in a bin containing distilled water and reached the laboratory within 20 minutes. The stems of spikes were recut about 2″ from the lower side in flowing distilled water to escape from air embolism. In glass vases, containing deionized distilled water, individual spikes were put while vase opening was wrapped with aluminum foil to control vase water evaporation. These spikes were carefully put in the laboratory tables at 26 ± 2°C with a relative humidity of about 70 ± 5%. The vase life of nine spikes per treatment was estimated by providing a 12-hour light duration with fluorescent lamps. The vase’s life was ended when the petals of the flower had symptoms of wilting, color fading, and petal abscission > 50%. On a daily basis, data was collected.

### 2.12 Statistical analysis

The obtained data were analyzed statistically by analysis of variance (ANOVA) technique by using STATISTICS (computer software program 8.1). The treatment means were compared by using the least significant difference test at a probability level of 5%. Principal component analysis and correlation matrix of different morpho-physiological and biochemical attributes of *Freesia refracta* due to supplementations of Si-NPs and IBA under Cr toxicity were assessed using version 2024 of OriginPro Software (Origin Lab Corporation, Northampton, MA, USA).

## 3 Results

### 3.1 Influence of Si-NPs and IBA on growth attributes under Cr stress

**Figure 1:**
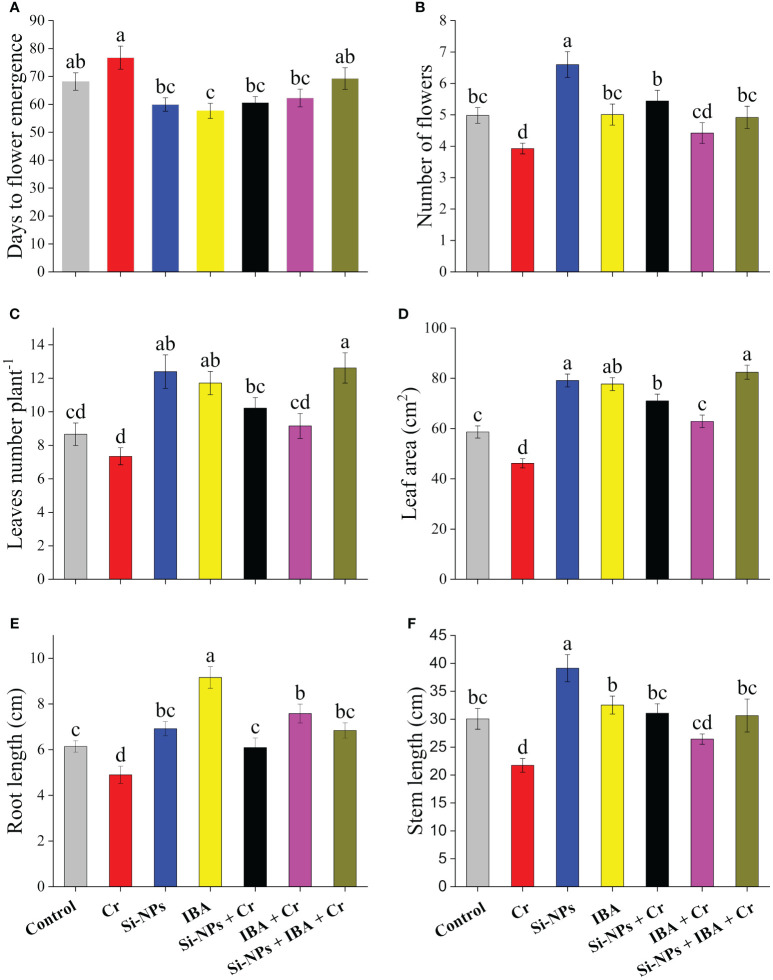
The Si-NPs and IBA impact on days to flower emergence **(A)**, flower numbers **(B)**, leaves numbers plant^-1^ **(C)**, leaf area **(D)**, root length **(E)**, and stem length **(F)** of *Freesia refracta* under Cr stress. Each bar illustrates the mean of five replications (n = 5). Dissimilar letters on bars represent significantly different (*P* ≤ 0.05).

It was observed that exposure of *Freesia refracta* to Cr stress significantly (*P* < 0.01) reduced growth attributes. Plants treated with Si-NPs and IBA produced flowers earlier (8.2 and 10.5 days respectively) than plants grown under control treatment, while Cr-stressed plants took more time (8.6 days) to produce flowers. Application of Si-NPs and IBA under Cr-stressed conditions resulted in earlier flowering (16 and 14.4 days respectively) compared to the untreated Cr-exposed plants (**Figure 1A**). The number of flowers per stem decreased by 21.1% under Cr-polluted conditions. Si-NPs and IBA supplementation increased flowering by 32.5% and 0.5% respectively, in unstressed plants compared with control plants. Meanwhile, a 38.5% and 12.5% increment in flower yield under Cr-stressed plants was observed with the exogenous spray of Si-NPs and IBA, respectively. The combined Si-NPs + IBA supplementation under Cr-stressed conditions, improved flower yield by 25.2% compared to untreated Cr-stressed freesia plants. Moreover, the number of leaves and leaf area of freesia plants were significantly reduced by 15.2% and 21.2%, respectively, under Cr stress. The supplementation of Si-NPs and IBA enhanced leaf numbers by 39.2% and 24.7%, respectively, while, leaf area increased by 53.8% and 36.2%, respectively, under Cr-stressed conditions. Both leaf parameters were also improved by 43.2% and 35.3% (leaf numbers) as well as 34.9% and 32.5% (leaf area) with foliar spray of Si-NPs and IBA respectively (**Figures 1B–D**). The data showed that Cr stress resulted in a decrease in length by 20.2% and 27.7% in the roots and stems of freesia plants, respectively, compared to the control. However, after the addition of Si-NPs and IBA, the length was enhanced by 12.7% and 49.2% in roots and 30.2% and 8.3% in stems respectively. Additionally, when Si-NPs and IBA were supplemented under Cr stress, an increment in the length of plants was recorded, reaching 24.3% and 54.7% in roots and 42.8% and 21.6% in stems of freesia plants respectively. Furthermore, data also revealed that the synergistic impact of Si-NPs + IBA was more profound in alleviating Cr toxicity than their respective individual treatments (**Figures 1E, F**).

### 3.2 Impact of Si-NPs and IBA on root and stem biomass under Cr toxicity

**Figure 2:**
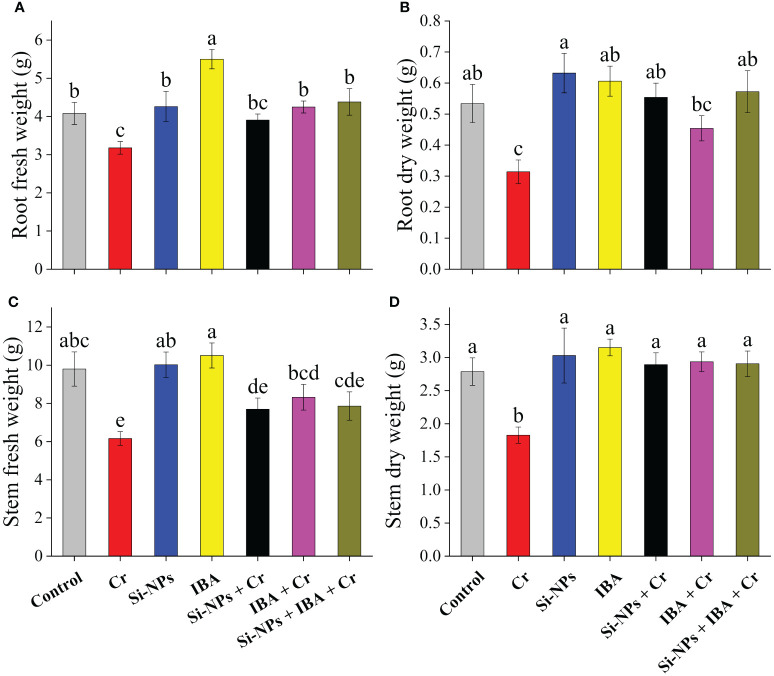
The Si-NPs and IBA impact on root fresh weight **(A)**, root dry weight **(B)**, stem fresh weight **(C)**, and stem dry weight **(D)** of *Freesia refracta* under Cr stress. Each bar illustrates the mean of five replications (n = 5). Dissimilar letters on bars represent statistically significant difference (*P* ≤ 0.05).

Statistically, both the fresh and dry biomass of roots and stems under Cr stress were significantly (*P* < 0.01) reduced in freesia plants. There was a 22.1% and 37.2% reduction in fresh biomass of roots, while a 41.2% and 34.3% decrease in dry biomass of stems was recorded under Cr-stressed conditions. The foliar spray of Si-NPs and IBA increased fresh biomass by 4.4% and 34.8% in roots and 2.3% and 7.2%, respectively, in stems of freesia plants. Similarly, dry biomass was increased by 18.4% and 13.5% in roots, while it increased by 8.7% and 13.1% in stems after adding Si-NPs and IBA, respectively, in unstressed plants compared to the control. Under Cr-stressed conditions, Si-NPs and IBA supplementation increased fresh root biomass by 22.8% and 33.7%, whereas fresh stem biomass increased by 24.9% and 35.1%, respectively, in comparison with Cr-stressed plants. The combined supplementation of Si-NPs + IBA to Cr-polluted freesia plants showed a more noticeable reduction in the antagonistic impact of Cr stress (**Figures 2A–D**).

### 3.3 Effect of Si-NPs and IBA on photosynthetic pigments under Cr-stress

**Figure 3:**
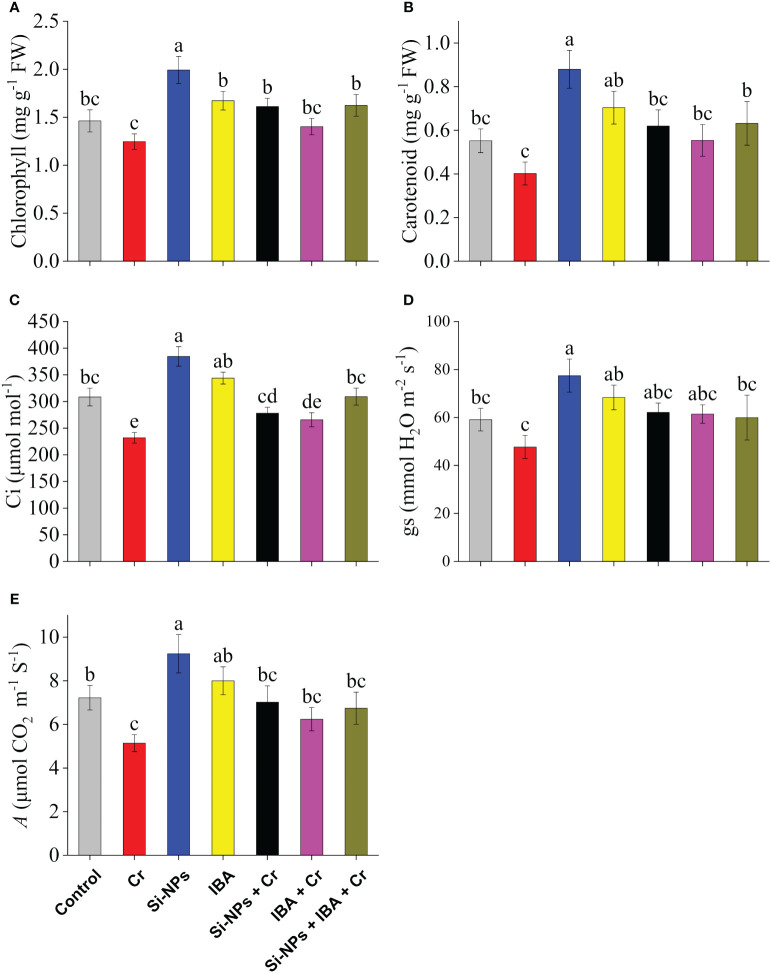
The Si-NPs and IBA impact on contents of chlorophyll **(A)**, carotenoid **(B)**, intercellular CO_2_ concentrations **(C)**, stomatal conductance **(D)**, and photosynthetic rate **(E)** of *Freesia refracta* under Cr stress. Each bar illustrates the mean of five replications (n = 5). Dissimilar letters on bars represent statistically significant difference (*P* ≤ 0.05).

Photosynthetic pigments such as chlorophyll and carotenoid contents in leaves of freesia were significantly (*P* < 0.01) reduced under Cr-stressed conditions by 14.7% and 27.2% respectively in comparison to control. The application of Si-NPs and IBA remarkably enhanced levels of both photosynthetic efficiency attributes under Cr-stressed and unstressed conditions compared with their respective treatments. The combined supplementations of Si-NPs + IBA under Cr stress showed a synergistic regulatory effect on chlorophyll levels by 30.3% and carotenoid contents by 57.1%, in comparison to Cr-exposed untreated plants (**Figures 3A, B**).

### 3.4 Impact of Si-NPs and IBA on gas exchange attributes under Cr-stress

Under Cr toxicity, *Ci* was reduced by 24.8%, *gs* by 19.3%, while *A* was decreased by 28.8% compared with the control. When Si-NPs and IBA were supplemented to Cr-exposed plants, the levels were increased by 36.5% and 21.4% in *Ci*, by 30.2% and 28.8% in *gs*, whereas, by 19.8% and 14.6% in *A*, respectively, compared with Cr-stressed untreated plants. A similar trend was also noted in plants supplemented with Si-NPs and IBA without the Cr stress regime. Likewise, under Si-NPs + IBA + Cr treatment, the augmentation of studied gas exchange attributes was enhanced by decreasing the lethal Cr impact on freesia plants compared to plants with Cr-stressed treatment (**Figures 3C–E**).

### 3.5 Effect of Si-NPs and IBA on proline and total protein level under Cr-stress

**Figure 4:**
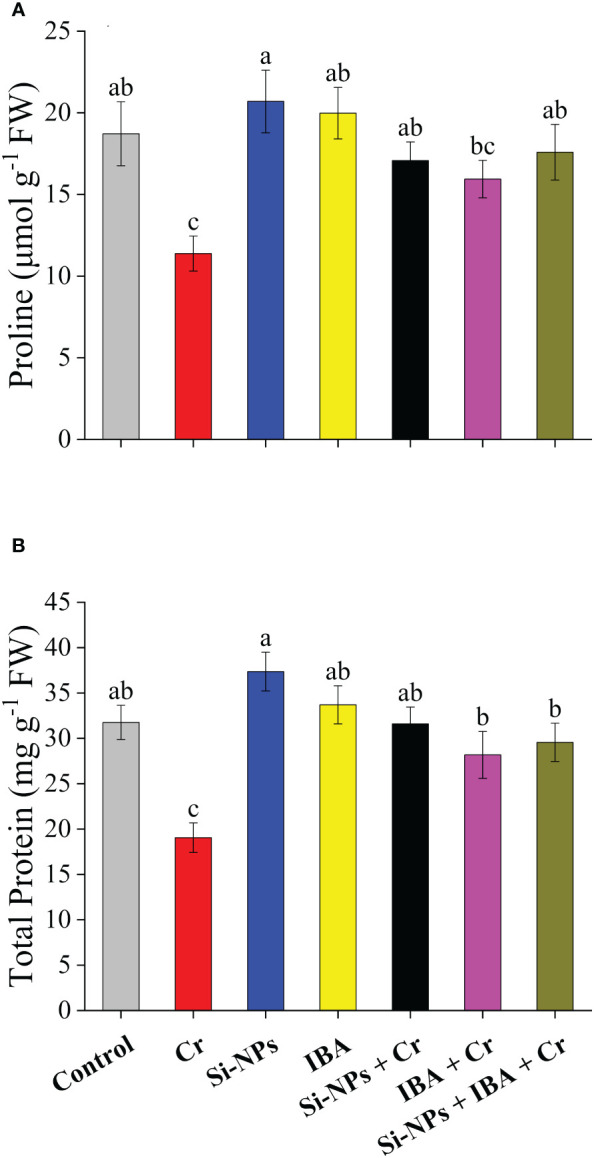
The Si-NPs and IBA impact on contents of proline **(A)**, and total protein **(B)** of *Freesia refracta* under Cr stress. Each bar illustrates the mean of five replications (n = 5). Dissimilar letters on bars represent statistically significant difference (*P* ≤ 0.05).

Statistically highly significant (*P* < 0.01) reducing impact was found under Cr stress for proline and total protein contents in freesia plants compared to the control. There was a reduction of 39.2% and 40% in proline and total protein contents, respectively, under Cr toxicity. Foliar spray of Si-NPs increased proline levels by 10.6% and 50.1% in unstressed and Cr-stressed plants respectively, whereas, IBA application enhanced proline levels by 6.7% and 40% in freesia plants under unstressed and Cr-exposed conditions respectively (**Figure 4A**). A similar trend was also recorded for total protein concentrations after exogenous foliar supplementations of Si-NPs+IBA. The integrated impact of Si-NPs + IBA under Cr toxicity, increased proline by 54.5% and total protein by 55.1% compared with Cr-stressed untreated freesia plants (**Figure 4B**).

### 3.6 Impact of Si-NPs and IBA on antioxidant enzyme activity under Cr stress

**Figure 5:**
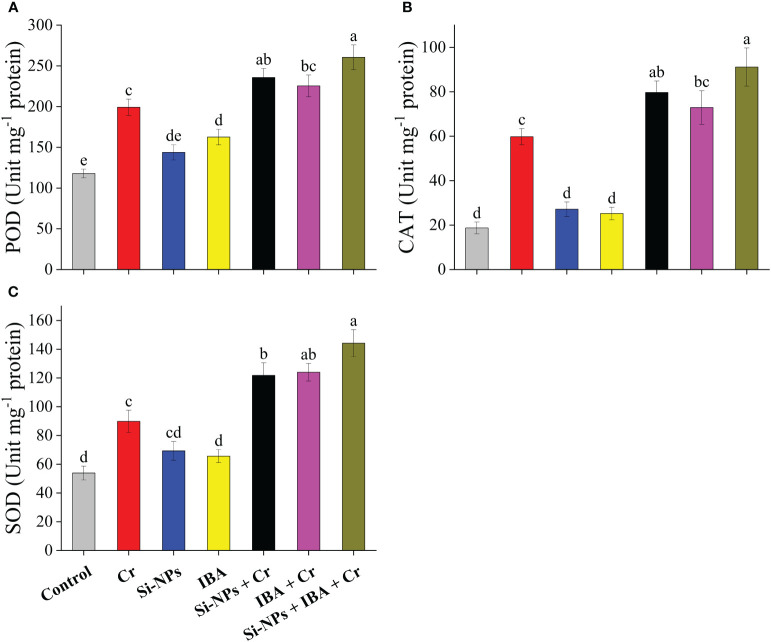
The Si-NPs and IBA impact on activities of peroxidase **(A)**, catalase **(B)**, and superoxide dismutase **(C)** of *Freesia refracta* under Cr stress. Each bar illustrates the mean of five replications (n = 5). Dissimilar letters on bars represent statistically significant difference (*P* ≤ 0.05).

The contents of POD, CAT, and SOD were significantly (*P* < 0.01) enhanced by 69%, 219.4%, and 66.5%, respectively, in freesia plants under Cr-stressed conditions compared to the control. The improved concentrations of these antioxidant enzymes under the alone as well as the combined spray of Si-NPs and IBA were helpful for plant growth and development. When the combined treatment of Si-NPs + IBA was given to freesia plants under Cr-stressed conditions, an increment of 30.8%, 52.4%, and 60.6% were recorded for POD, CAT, and SOD, respectively. A similar trend was also observed when Si-NPS and IBA were individually supplemented under both Cr-stressed and unstressed conditions (**Figures 5A–C**).

### 3.7 Influence of Si-NPs and IBA on MDA and H_2_O_2_ under Cr stress

**Figure 6:**
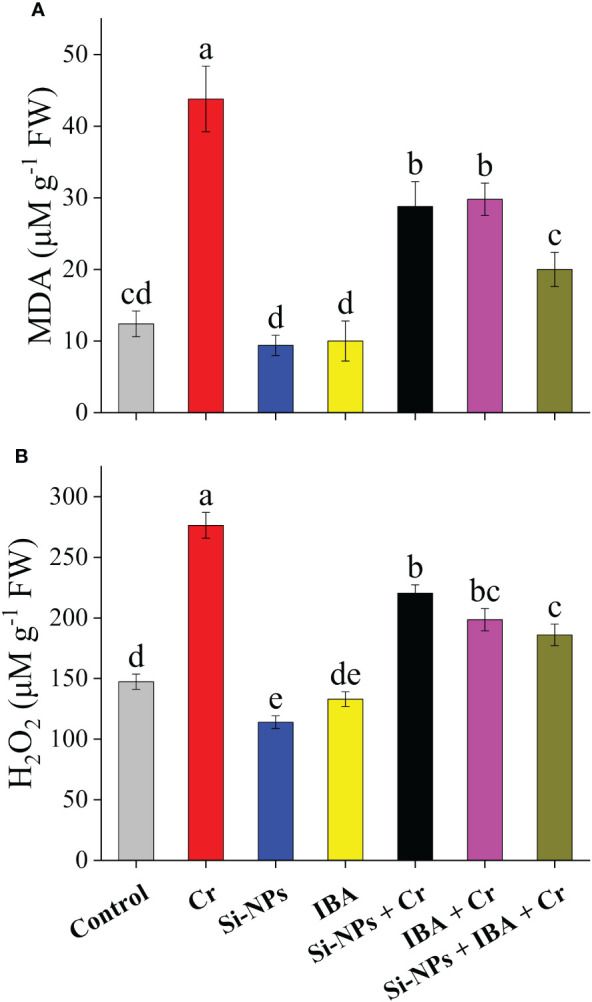
The Si-NPs and IBA impact on contents of malondialdehyde **(A)**, and hydrogen peroxide of **(B)** *Freesia refracta* under Cr stress. Each bar illustrates the mean of five replications (n = 5). Dissimilar letters on bars represent statistically significant difference (*P* ≤ 0.05).

Exposure of freesia plants to Cr stress significantly (*P* < 0.01) increased MDA and H_2_O_2_ levels by 253.2% and 87%, respectively, compared to the control. Foliar application of Si-NPs and IBA reduced MDA by 24.1% and 22.7%, while, H_2_O_2_ was reduced by 19.4% and 9.7%, respectively, in unstressed plants (**Figures 6A, B**). Similarly, Cr-stressed plants showed a reduction in MDA and H_2_O_2_ levels by 34.2% and 20.3% with Si-NPs spray, while with IBA supplementation, the reduction was 32% and 28.1%, respectively. However, the SiNPs + IBA + Cr treatment proved to be more efficient, decreasing the level of MDA by 54.3% and H_2_O_2_ by 32.7% compared to Cr-stressed untreated plants (**Figures 6A, B**).

### 3.8 Impact of Si-NPs and IBA on the uptake and translocation of Cr in various plant parts

**Figure 7:**
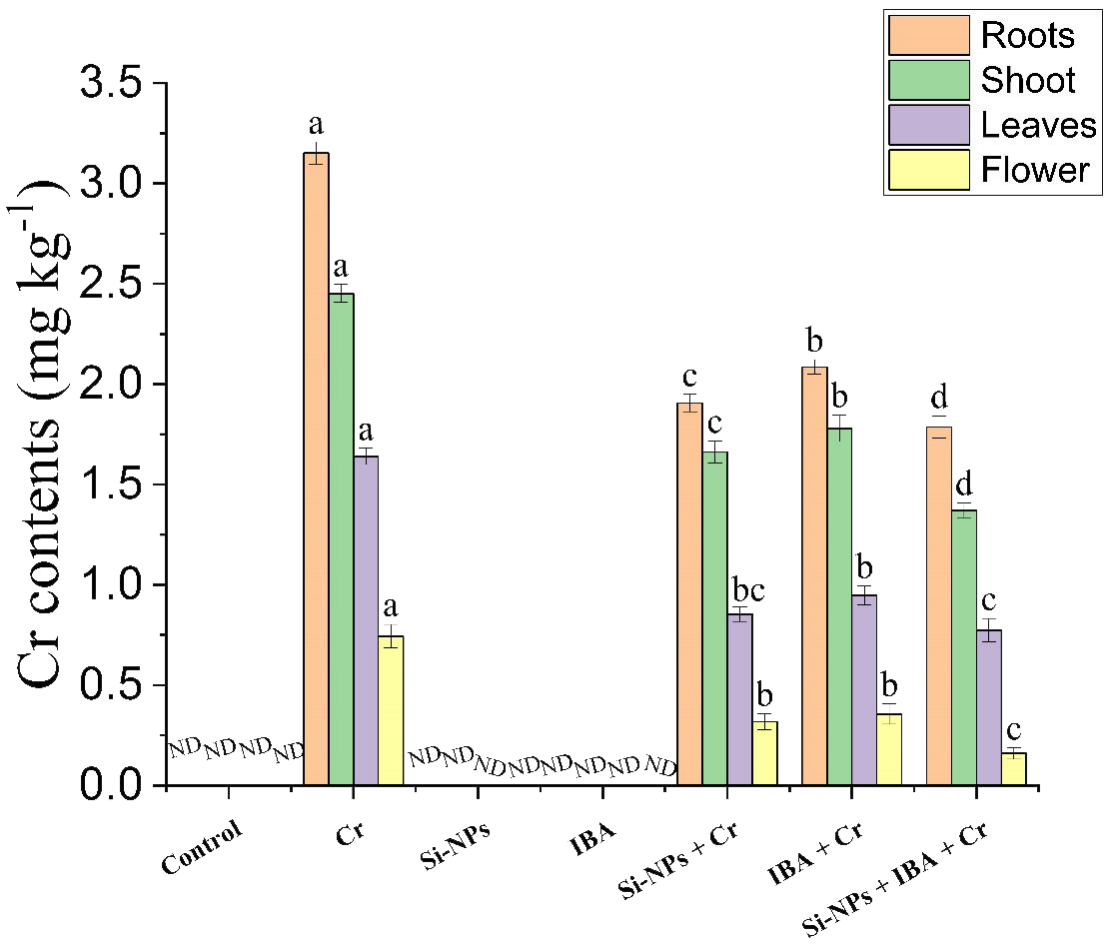
The Si-NPs and IBA impact on Cr uptake and translocation different plant parts of *Freesia refracta* under Cr stress. Each bar illustrates the mean of five replications (n = 5). Dissimilar letters on bars represent statistically significant difference (*P* ≤ 0.05). ND, Not detected.

Experimental findings regarding the contents of Cr demonstrated that both Si-NPs and IBA treatments remarkably reduced the uptake of Cr in freesia plants from soil to roots and its translocation to shoots, leaves, and flowers under Cr toxicity. Freesia plants cultivated on Cr-polluted soils showed the greatest Cr accrual in all studied plant tissues compared to other experimental treatments. However, foliar supplementation of Si-NPs reduced Cr contents by 39.5%, 32.2%, 47.9%, and 57.3%, whereas, IBA application abridged Cr levels by 33.8%, 27.4%, 42.2%, and 52.2%, in roots, stems, leaves, and flowers, respectively, in comparison to untreated Cr-stressed plants. Furthermore, the combined application of Si-NPs + IBA also reduced Cr stress more efficiently than individual treatments by 43.3%, 44%, 52.8%, and 78.5% in roots, stems, leaves, and flowers respectively. Experimental data also suggested that the highest Cr contents were found in roots followed by stems, leaves, and flowers respectively (**Figure 7**).

### 3.9 Impact of Si-NPs and IBA on vase life of freesia

**Figure 8:**
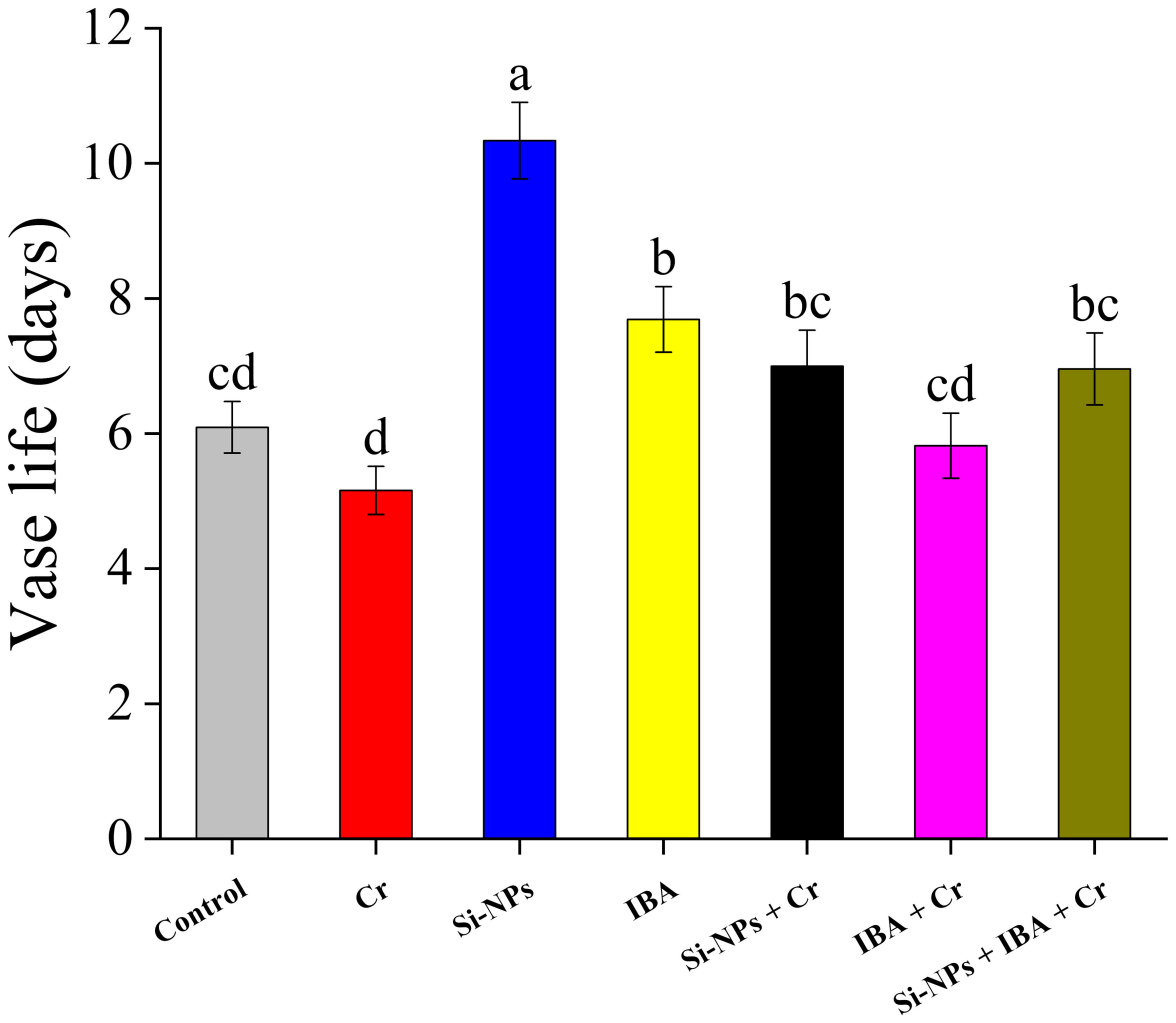
The Si-NPs and IBA impact on vase life of *Freesia refracta* under Cr stress. Each bar illustrates the mean of five replications (n = 5). Dissimilar letters on bars represent statistically significant difference (*P* ≤ 0.05).

The vase life of Cr-stressed plants was shorter (15.3%) compared to the control. The application of Si-NPs and IBA alone, without Cr-stressed conditions, significantly enhanced the vase life of freesia cut flowers by 69.7% and 26.2%, respectively, compared to the control. Under Cr-exposed conditions, Si-NPs and IBA alone, as well as with combined treatment, also moderately increased vase life but were less effective compared to unstressed conditions (**Figure 8**).

### 3.10 Principal component analysis and heat map Pearson correlation

**Figure 9:**
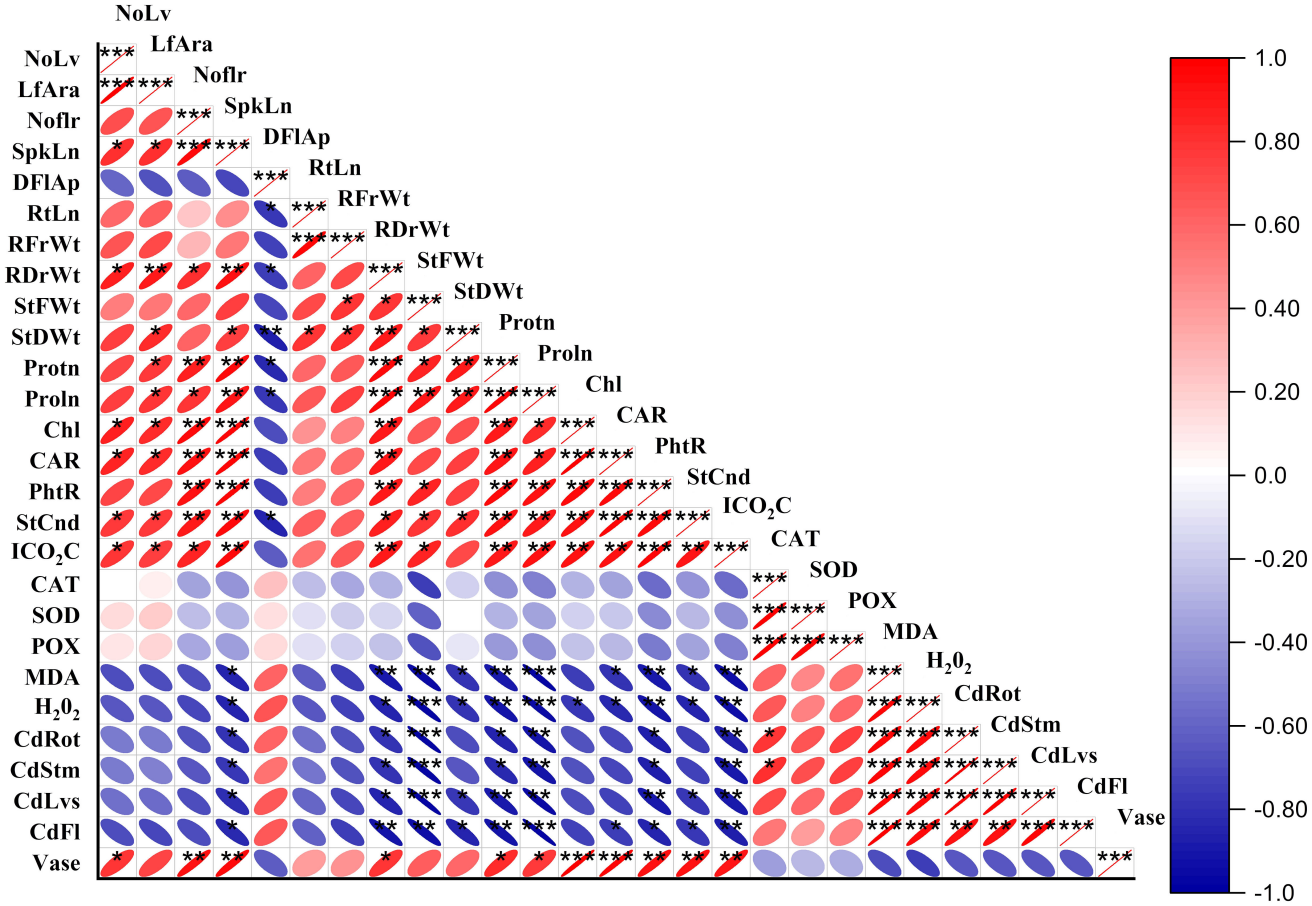
Heat map Pearson correlation coefficients on morphological, physiological and biochemical attributes of freesia plant under Cr stress. NoLf, Numbers of leaf; LfAra, Leaf area; NoLf, Number of flowers; SpkLn, Spike length; DFIAp, Days to flowers; RtLn, Root length; RFrWt, Root fresh weight; RDrWt, Root dry weight; StFWt, Stem fresh weight; StDWt, Stem dry weight; Protn, Protein; Proln, Proline; Chl, Chlorophyll; CAR, Carotenoid; PhtR, Photosynthetic rate; StCnd, Stomatal conductance; ICO2C, Intercellular CO_2_ concentrations; CAT, Catalase; SOD, Superoxide dismutase; POX, peroxidase; MDA, Malondialdehyde; H2O2, Hydrogen peroxide; CdRot, Cadmium in roots; CdStm, Cadmium in stems; CdLvs, Cadmium in leaves; CdFl, Cadmium in flowers; Vase, Vase life. *P ≤ 0.05; **P ≤ 0.01; ***P ≤ 0.001.

The heat map in **Figure 9** displays Pearson correlation coefficients among various growth and biochemical parameters of *Freesia refracta* under Cr stress. Significant positive correlations were observed among several growth attributes. However, Cr treatment had adverse effects on root length (RtLn), root fresh weight (RFrWt), and stem fresh weight (StFWt), as indicated by the negative correlations (**Figure 9**). The antioxidant enzymatic activities in *Freesia refracta* under Cr toxicity also showed a combination of positive and negative correlations. This heat map shows the relationships and statistical significance between the measured parameters using the average values from the study. The color gradient represents the strength and direction of the correlations, with red indicating positive correlations and blue indicating negative correlations.

**Figure 10:**
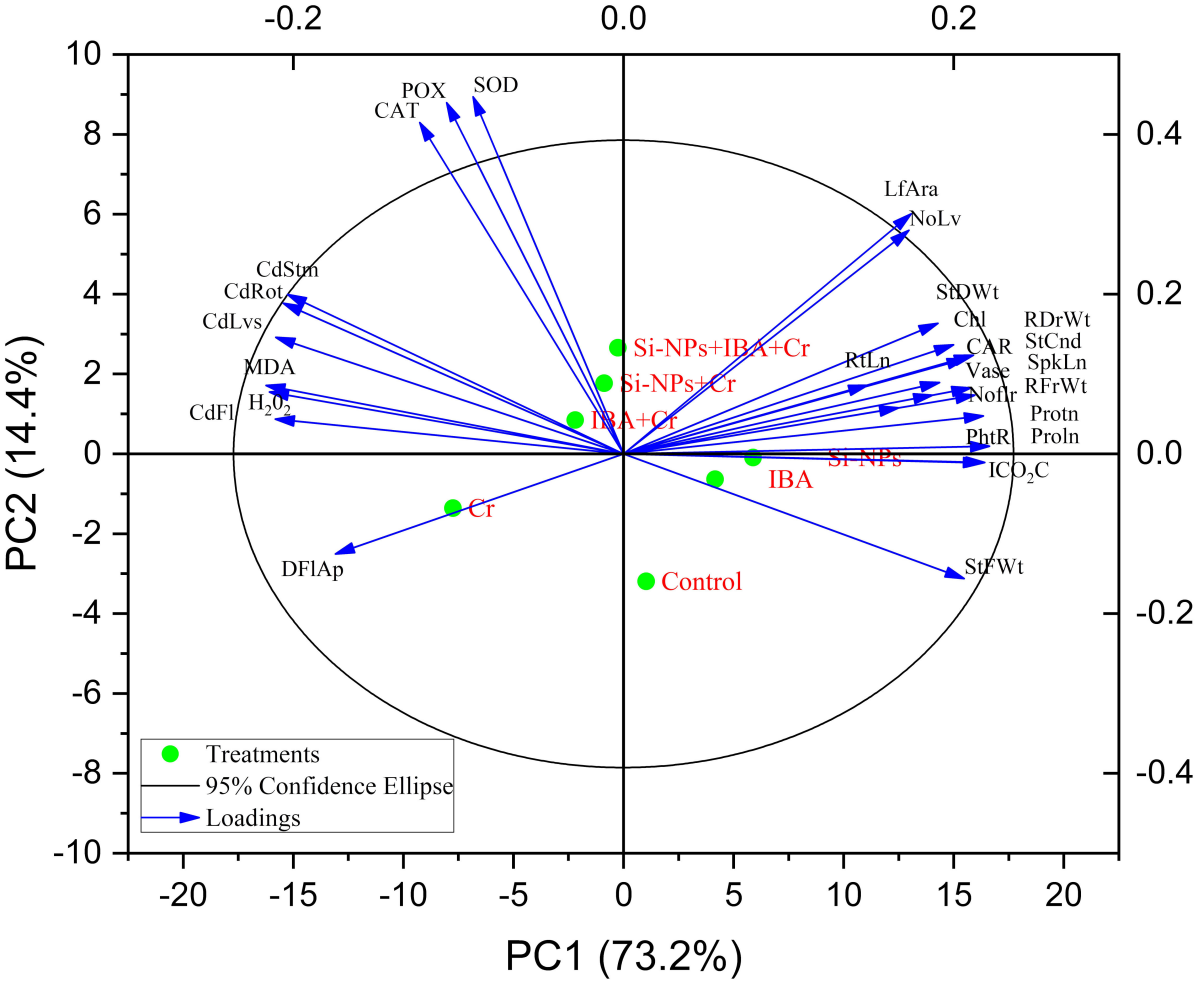
Principal component analysis biplots showing correlation among various morphological attributes of freesia plant as influenced by alone and combined application of silicon nanoparticles and indole-3-butyric Acid (IBA) treatments under Cr stress conditions. NoLf, Numbers of leaf; LfAra, Leaf area; NoLf, Number of flowers; SpkLn, Spike length; DFIAp, Days to flowers; RtLn, Root length; RFrWt, Root fresh weight; RDrWt, Root dry weight; StFWt, Stem fresh weight; StDWt, Stem dry weight; Protn, Protein; Proln, Proline; Chl, Chlorophyll; CAR, Carotenoid; PhtR, Photosynthetic rate; StCnd, Stomatal conductance; ICO2C, Intercellular CO_2_ concentrations; CAT, Catalase; SOD, Superoxide dismutase; POX, peroxidase; MDA, Malondialdehyde; H2O2, Hydrogen peroxide; CdRot, Cadmium in roots; CdStm, Cadmium in stems; CdLvs, Cadmium in leaves; CdFl, Cadmium in flowers; Vase, Vase life.

In the PCA results shown in **Figure 10**, the eigenvalues and contributions of PC1 and PC2 are displayed, explaining 87.6% of the variability in the data set, with PC1 contributing 73.2% and PC2 contributing 14.4%. The PCA separated the response variables based on the relationship between the input and response variables, particularly in the Si-NPs and IBA treatments of *Freesia refracta* under Cr stress. IBA treatment was associated with improvements in photosynthetic pigments and gas exchange attributes. Enzyme activities of POD, CAT, and SOD in *Freesia* plants increased under Cr-stressed conditions and were linked to the combined application of Si-NPs + IBA. The use of Si-NPs and IBA significantly enhanced photosynthetic efficiency characteristics under both Cr-stressed and unstressed conditions when compared to their respective control treatments. MDA and H_2_O_2_ were classified together due to their similar reactive nature. Applied Cr stress (IBA + Cr) negatively affected the levels of H_2_O_2_ and MDA in *Freesia* plants. Furthermore, **Figure 10** illustrates that the uptake of Cr from the soil into the roots, shoots, leaves, and flowers of *Freesia* plants is inversely correlated with Si-NPs and IBA treatments under Cr-stressed conditions.

## 4 Discussion

It has been observed by numerous studies that nanoparticles play a key role in enhancing plant growth and ameliorating heavy metal stress (Li et al., 2021). Indole butyric acid (IBA) is an analog of indole acetic acid (IAA), it has a main role in plant responses to development and abiotic stressors (Ran et al., 2018). However, the role of Si-NPs along with IBA to minimize antagonistic heavy metal impacts on ornamental geophytes such as freesia, in particular, Cr-mediated oxidative damage was not investigated. Harmful effects on vegetative and reproductive growth attributes are major gages of Cr-induced stress in plants. The present experiment showed delayed flowering of freesia plants, while, foliar spray of Si-NPs and IBA combined and individually reduced production time and early flowering. These results were parallel with the findings of Ahmad et al. (2019), who explained that growth promotors with optimum concentrations may result in early flowering by encouraging the growth rate, improving early leaf maturation, increasing flowering yield, and greater production of photosynthate. Heavy metal stress, as speculated in this experiment, reduced morphological growth and development of freesia. This reduction in growth may be owing to Cr due to reduced water and nutrient absorption and transportation in plants (Vezza et al., 2022). The Cr-mediated growth retardation also be the result of abridged photosynthetic efficacy and sugar metabolism (Ayyaz et al., 2021). Additionally, a changed hormonal balance in response to Cr toxicity could be an important cause for stunted morphological growth in plants grown under Cr-stressed conditions (Naaz et al., 2023). The present study indicated that the application of Si-NPs and IBA ameliorated the damaged vegetative and reproductive growth parameters caused by Cr stress (**Figures 1A–D**). The use of Si-NPs and auxins had a positive influence in increasing leaf area and flower numbers of *Freesia refracta* and hence lessened Cr-mediated phytotoxic impacts. These results were in accordance with the findings of Wang et al. (2022); Naaz et al. (2023); Rahman et al. (2023), and Siposova et al. (2021) on different crops. They reported that plant vegetative traits were noticeably increased by the supplementation of Si-NPs and IBA in comparison with control by reducing the toxicity due to Cr stress. As IBA are commonly used for the stimulation of root growth of stem cuttings (Tworkoski and Takeda, 2007), this study also found longer roots and shoots than other treatments (**Figures 1E, F**). Similar findings were obtained in *Psidium guajava* (Shahzad et al., 2019) and *Euclyptus* species (Fogaça and Fett-Neto, 2005) which concluded that IBA is efficient due to the slow and continuous release of indole acetic acid (IAA) from IBA. Root elongation is promoted with IBA due to influencing the enzyme synthesis involved in cell enlargement and increasing the translocation of carbohydrates to the root zone (Ali et al., 2009). Biomass of roots and stems was significantly enhanced by the treatments of Si-NPs and IBA alone and in combination under Cr-treated and untreated conditions. Similarly, Sharma et al. (2022) stated that Si-NPs and auxins had a positive impact in increasing the fresh and dry biomass of rice seedlings and thus ameliorating the lethal impacts of Cr toxicity.

Photosynthetic pigments and gas exchange indices are efficient physiological gages for evaluating the intensity of plants cultivated under abiotic stress (Ahsan et al., 2023). The present study showed a remarkable reduction in chlorophyll concentrations, carotenoids, and gas exchange attributes in plants cultivated in Cr-polluted soils, leading to the production of lesser plant biomass. On the other hand, supplementation of Si-NPs and IBA in Cr-treated plants assisted in incapacitating the reduced photosynthetic pigments and contents of gas exchange attributes (**Figures 3A–E**). Initial impacts of Cr toxicity on photosynthetic apparatus comprised of changes in the ultrastructure of chloroplast, decreased rubisco activity, alteration in the composition of pigments, abridges photosynthetic rate and stomatal conductance, and inhibition in activities of electron transport chain (Rahman et al., 2023). Similarly, Ahmad et al. (2023) stated that Si-NPs enhanced the photosynthetic pigments and gas exchange attributes in wheat plants under Cr stress. Chen et al. (2024) reported that IBA treatment may considerably enhance photosynthetic pigments under heavy metal stress by increasing Mg contents in plant leaves which decreased the possibility that heavy metals would replace central Mg in the chlorophyll. Generally, increased chlorophyll concentration results in elevated photosynthesis and improved performance of the plants (Altaf et al., 2022). Furthermore, the stabilization of photosynthetic pigments and total photosynthesis is associated with the reduction of oxidative injury and Cr absorption after foliar spray of Si-NPs and IBA (Adrees et al., 2022; Barzin and Firozabadi, 2023; Chen et al., 2024). Siposova et al. (2021) reported that IBA increased the electron flow of around the PS-I and PS-II by increasing the electron transport rate in PS-I and PS-II. Si-NPs and exogenous auxin synergistically improved the efficiency of photosynthesis and decreased photosynthetic mechanism disturbance due to Cr-toxicity (Sharma et al., 2022).

Proline has a regulatory role in osmotic variations, membrane balance, and excessive ROS abolition, which controls plant survival under changing environmental situations (Ghosh et al., 2022). Generally, its accumulation plays a remarkable role in regulating the development of plants under stressed conditions (Fatnani et al., 2023). Under abiotic stress, proline is formed in all plants with varied concentrations and acts as an operative ROS quencher (Raklami et al., 2021). The present study showed elevated proline levels in plants treated with Si-NPs and IBA alone and in combination under Cr-stressed and unstressed conditions (**Figure 4A**). Endogenous proline accrual in plants during stress conditions guard plants against ROS-mediated injury via recovering the bothered osmotic balance (Jan et al., 2023). Very similar to our results, the addition of nanoparticles under Cr-stressed maize plants significantly enhanced proline accrual (Ramzan et al., 2023). Furthermore, the concentration of protein may be an ideal indicator to measure the physiological state of the plant. Cr toxicity reduced the protein level in this study. It has been proposed to findings of reduced protein synthesis under lethal Cr stress, and increased protease activity disturbs the protein structure and activity, which in turn results enhanced extent of protein denaturation (Faizan et al., 2021). Sharma et al. (2022) reported that exogenous auxin (IBA) application enhanced the hormone levels in plants resisted the lethal effects caused by heavy metals and prevented growth inhibition. This study proved the findings of Sharma et al. (2022) that the application of Si-NPs and IBA lessens Cr-mediated protein degradation in the freesia plants, as has been described in this experiment. Plant growth is deeply dependent on the synthesis of protein, while Si-NPs and IBA treatments individually and synergistically increase the protein concentrations under Cr-stressed conditions (Rahman et al., 2023). The precise underlying process that guides this mechanism is not properly understood but it is supposed that there is a strong correlation between Si-NPs and protein under Cr toxicity.

Activities of antioxidant enzymes work together against oxidative stress to defend plants from the dangers of heavy metal stress (Zhu et al., 2022). Plants have possessed some outstanding defense antioxidant enzyme activities like POD, CAT, and SOD to normalize oxidative damage and diminish the accumulation of ROS (Priyanka et al., 2021). Prominently, the damaging impact of Cr stress was alleviated by using Si-NPs and IBA individually and in combination treatment, which noticeably enhances the antioxidant enzyme activities in freesia plants. Previous studies found that SOD changes O_2_^∺−^ (which is greatly harmful) into H_2_O_2_ (which is lesser harmful) followed by conversion to O_2_ by CAT. H_2_O_2_ is further decomposed by POD through a circle cycle of ascorbate-glutathione and protects the plants against oxidative stress (Ahmad et al., 2023). The activities of antioxidant enzymes are further enhanced by the application of Si-NPs and IBA under Cr stress, which protects freesia plants more efficiently against Cr toxicity (**Figure 5**). The IBA supplementation alleviates the heavy metal stress in two possible pathways. The first possible pathway is that IBA might have scavenged the Cr in the cytoplasm as auxin can act as a chelating agent (Saini et al., 2021) because IBA, in the cytoplasm, presents in the deprotonated form that has a large affinity to heavy metals. The second possible pathway is that foliar treatment of IBA probably enhancements the decreased internal auxin contents, which can affect the levels of signaling molecules and can be involved in the pathways of signaling defense mechanisms e.g., biosynthesis of glutathione, metal-binding ligands, and phytochelatins (Singh et al., 2021). However, the response of Si-NPs and IBA on antioxidant enzyme activities is complicated and differs with the kind of metal toxicity, species of the plant, stages of growth, duration of HM exposure, and Si-NPs and IAA method of application (Hussain et al., 2019). The findings of this study showed that contents of MDA and H_2_O_2_ increased remarkably when Cr stress was imposed in freesia plants (**Figure 6**). Lipid peroxidation induced by heavy metal toxicity in terms of MDA concentration and oxidative injury, which results in accrual of ROS such as H_2_O_2_ which harshly damage the organelles and plant cells functioning (Bhattacharjee et al., 2022). However, Si-NPs and IAA can decline HM stress by decreasing lipid peroxidation, as shown by a reduction in MDA and H_2_O_2_ (Alam et al., 2022) as compared with control. Foliar supplementation of Si-NPs decreased the ROS level and regulated the stability of the membrane by recovering the cell membrane damage to Cr-stressed plants (Dai et al., 2021). Sharma et al. (2022) reported that the entry of Cr ions into the plant cell disturbs the membrane integrity and structure of cells which leads to ROS overproduction whereas, Si-NPs and auxin ameliorated these drastic impacts resulting in the maintenance of cell membrane integrity.

The concentration of Cr was estimated by measuring the Cr contents in roots, shoots, leaves, and flowers of freesia plants. Results of this study showed reduced Cr uptake and root-to-flower translocation after exogenous supplementation of Si-NPs and IBA (**Figure 7**). Parallel findings were documented by Mohammadi et al. (2020), who showed that FeONPs significantly reduced Cr accrual and its translocation from root to higher plant parts in sunflower, and in studies with Si-NPs in pea (Tripathi et al., 2015) and in investigations with Cu-NPs in wheat (Noman et al., 2020). Ali et al. (2019) reported that foliar supplementation of Si-NPs greatly reduced toxic metal uptake in wheat roots to grains. The reduced Cr concentrations in plant parts by foliar spray of Si-NPs and IAA individually and with combined treatment may increase the binding of Cr to plant tissues and enhance the compartmentalization of Cr in plant vacuoles, which ultimately reduces Cr translocation towards reproductive parts of the plant (Sardar et al., 2022). Exogenous IBA supplementation ameliorates Cr-induced oxidative damage by reducing its accumulation in plant parts and improves Cr tolerance in Cr-stressed plants (Gangwar and Singh, 2011). Chen et al. (2022) reported that foliar supplementation of Si-NPs remarkably influenced the Cd concentrations in rice plants. The smallest Cr contents in reproductive plant parts by the Si-NPs supplementation might be because of the co-precipitation of metals with Si in less active plant parts metabolically (Huang et al., 2024).

**Figure 11:**
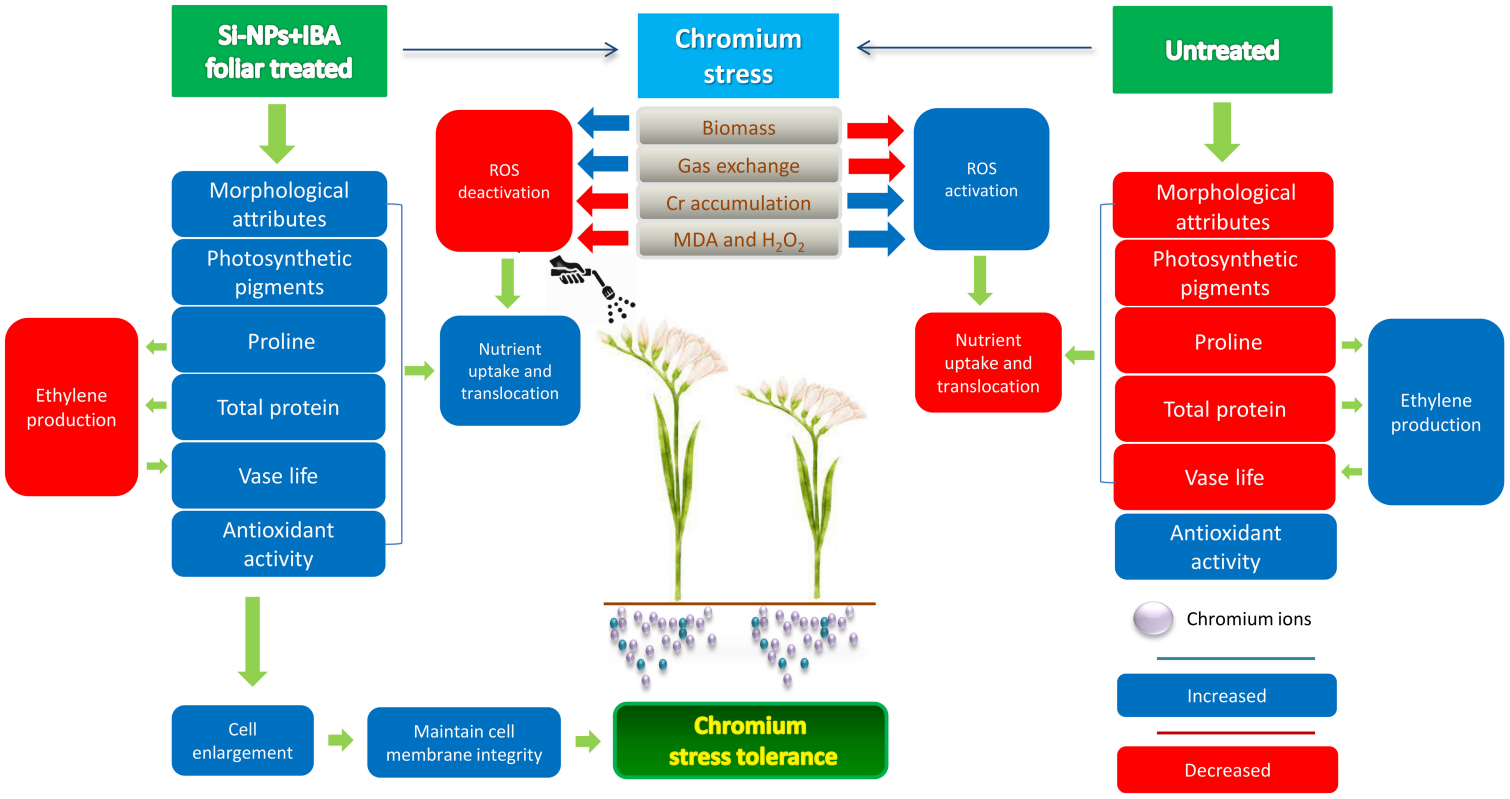
Mechanisms of Cr-stress tolerance in untreated and Si-NPs + IBA treated Freesia refracta plants. The simulation and inhibition of respective processes/attributes are indicated with blue color and red color tiles, respectively.

The vase life of freesia is slightly reduced by Cr stress without any treatment. Application of Si-NPs and IBA treatment individually and in combination improved the vase life with and without Cr stress (**Figure 8**). Enhancement in vase life of freesia plant due to Si-NPs and IBA application could be linked with improvement in protein concentration and photosynthetic pigments (**Figure 11**). Aier et al. (2017) stated that nanoparticles are more suitable for inhibiting ethylene production and increasing the vase life of rose cultivars. Previous studies also revealed that NPs remarkably decrease blockage of the xylem and inhibit ethylene production, thereby improving the vase life of cut flowers (Naing et al., 2017; Park et al., 2017). Janowska and Stanecki (2013) also reported that auxin supplementation improved sugar and proline in lily-cut flowers which resulted in an increased vase life of up to three days.

## 5 Conclusion

The current study investigated the efficient and effective roles of Si-NPs and IBA (individually and in synergistically) in ameliorating Cr stress in elegant freesia-cut flowering plants. This study proved that foliar supplementations of Si-NPs and IBA improved vegetative and reproductive attributes and root-stem biomasses. These positive stimulations were found to be associated with Si-NPs and IBA-mediated improvements in physiological mechanisms to reduce the ROS accrual, increase chlorophyll and gaseous exchange attributes, stimulate proline and total protein contents and improve efficiency of antioxidant enzymatic machinery as found in this experiment. These phenomena reduced Cr uptake and translocation in different plant parts which ultimately enhanced the vase life of *Freesia refracta*. Cr-stressed freesia plants significantly enhanced MDA, H_2_O_2_, and Cr contents in below and above ground plant parts, but the exogenous supplementation of Si-NPs and IBA might alleviated such antagonistic increments. This study proposed that Si-NPs (strongly impacted) and IBA (moderately impacted) individually and Si-NPs + IBA (strongly effective) synergistically have a positive regulatory impact on freesia plants under Cr-stressed conditions. The present experiment partially fills the gap by seeking the impact of Si-NPs and IBA (individually as well as synergistically) on the vase life elongation of freesia cut flower and the key physiological and biochemical modifications inducing growth performance under Cr-stressed conditions. Furthermore, comprehensive investigations are strongly suggested under several climatic conditions by utilizing different floricultural crops before the final recommendation regarding the use of Si-NPs individually and in combination with IBA to reduce the uptake and translocation of heavy metals in plants. It is also recommended to examine the impacts of Si-NPs and IBA under heavy metal toxicity conditions on multi-contaminated soil. In future experiments, the mechanisms by which NPs and auxins withstand Cr toxicity in ornamental geophytes must be further investigated using molecular techniques.

## Data availability statement

The original contributions presented in the study are included in the article/supplementary material. Further inquiries can be directed to the corresponding author.

## Author contributions

MA: Conceptualization, Data curation, Formal analysis, Methodology, Project administration, Writing – original draft, Writing – review & editing. ER: Conceptualization, Formal analysis, Project administration, Supervision, Writing – original draft, Writing – review & editing. AJ: Formal analysis, Resources, Validation, Visualization, Writing – original draft, Writing – review & editing. HA: Funding acquisition, Investigation, Methodology, Supervision, Writing – review & editing. MS: Investigation, Project administration, Resources, Writing – review & editing. AM: Data curation, Formal analysis, Project administration, Writing – review & editing. AB: Conceptualization, Data curation, Investigation, Methodology, Validation, Writing – review & editing. MN: Project administration, Resources, Validation, Writing – review & editing. JK: Data curation, Formal analysis, Resources, Writing – review & editing. MV: Data curation, Formal analysis, Investigation, Resources, Software, Writing – review & editing.
